# Nano-enabled bioanalytical approaches to ultrasensitive detection of low abundance single nucleotide polymorphisms

**DOI:** 10.1039/c4an02304h

**Published:** 2015-03-18

**Authors:** Lorico D. S. Lapitan Jr., Yuan Guo, Dejian Zhou

**Affiliations:** a School of Chemistry and Astbury Centre for Structural Molecular Biology, University of Leeds, Leeds LS2 9JT, UK. Email: d.zhou@leeds.ac.uk; b Department of Chemical Engineering, Faculty of Engineering, University of Santo Tomas, Espana Boulevard, Manila, Philippines

## Abstract

Single nucleotide polymorphisms (SNPs) constitute the most common types of genetic variations in the human genome. A number of SNPs have been linked to the development of life threatening diseases including cancer, cardiovascular diseases and neurodegenerative diseases. The ability for ultrasensitive and accurate detection of low abundant disease-related SNPs in bodily fluids (*e.g.* blood, serum, *etc*.) holds a significant value in the development of non-invasive future biodiagnostic tools. Over the past two decades, nanomaterials have been utilized in a myriad of biosensing applications due to their ability of detecting extremely low quantities of biologically important biomarkers with high sensitivity and accuracy. Of particular interest is the application of such technologies in the detection of SNPs. The use of various nanomaterials, coupled with different powerful signal amplification strategies, has paved the way for a new generation of ultrasensitive SNP biodiagnostic assays. Over the past few years, several ultrasensitive SNP biosensors capable of detecting specific targets down to the ultra-low regimes (*ca.* aM and below) and therefore holding great promises for early clinical diagnosis of diseases have been developed. This mini review will highlight some of the most recent, significant advances in nanomaterial-based ultrasensitive SNP sensing technologies capable of detecting specific targets on the attomolar (10^–18^ M) regime or below. In particular, the design of novel, powerful signal amplification strategies that hold the key to the ultrasensitivity is highlighted.

## Abstract 2

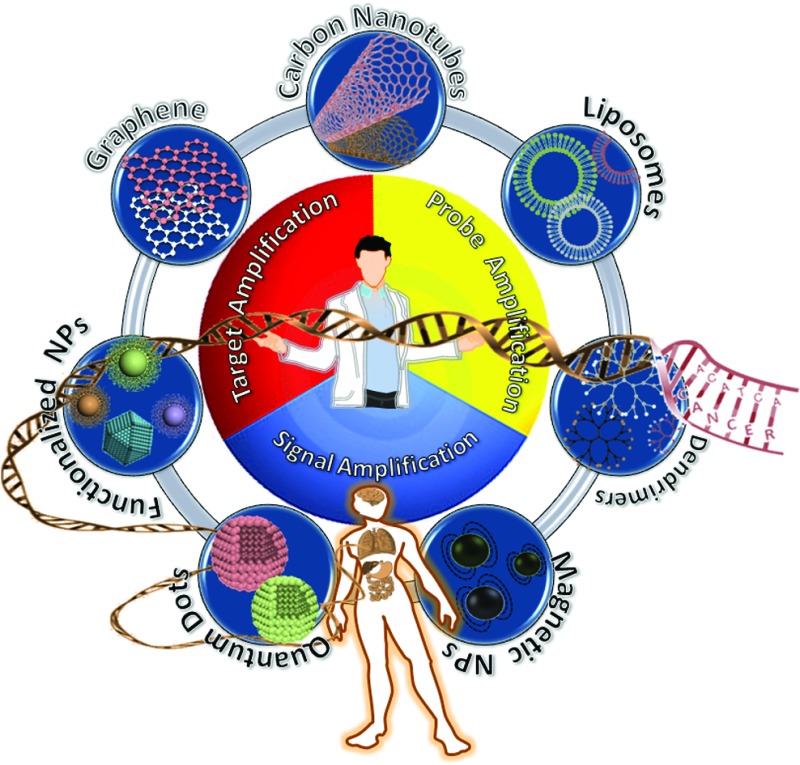
A survey of the recent, significant developments on nanomaterials enabled ultrasensitive DNA and gene mutation assays is presented.

## 1. Introduction

The completion of the human genome project in 2003^1^ has led to several important discoveries relating to structure of the human genome: it is characterized by variations in DNA nucleotide sequences of one or more bases in genes of the same population.^2,3^ These variations in DNA impart certain phenotypic traits that distinguish an organism from another. If such variations occur in greater than one percent of the human population, they are collectively referred to as polymorphisms. On the other hand, variations occurring in less than one percent of the population are often termed as “mutations” which are associated with a detrimental phenotype such as those linked with various cases of cancer.^3^ The most common sequence variation occurring in the human genome is the stable substitution of a single nucleotide, also known as single nucleotide polymorphisms (SNPs). The human genome has been estimated to contain more than 10 million SNPs which are distributed across the human genome at an estimated frequency of at least one nucleotide every 1000 base pairs but with apparent regional differences.^4,5^ While most SNPs do not alter the metabolic function and expression levels of a gene, some do result in differences in predisposition to certain heritable diseases,^6–8^ response to drugs (pharmacogenetics),^9,10^ and perception of pain.^11^ Several studies have shown that SNPs are closely associated with many types of cancer and presupposes the risk of cancer progression.^12–15^ For instance, it was observed that single point mutations in the KRAS gene at codons 12, 13, and 61 are associated with the development of certain pancreatic^14^ and lung cancers.^15^ Moreover, numerous detrimental and inheritable diseases such as diabetes,^7^ vascular diseases,^16^ some forms of neurodegenerative and mental illness^17,18^ have also been linked to SNPs. On the other hand, microRNAs (miRNAs) which are 17–24 base short RNA molecules playing important roles in numerous cellular processes such as differentiation, cellular growth, and apoptosis. Point mutations in miRNAs have been associated with several forms of cancers, affecting the cancer risk and treatment efficacy in some non-small cell lung cancer patients.^19,20^ These are just a few examples highlighting the association of SNPs in relation to human diseases and treatment responses, demonstrating the tremendous value of SNPs in biomedical research. Indeed, SNPs are considered as an important class of biomarkers that could allow scientists and medical practitioners to better understand certain diseases, develop novel non-invasive diagnostics tool and ultimately allow for a more personalized approach to disease treatment and therapies.

The most established techniques for point mutation detections in DNA have largely relied on the amplification power of the polymerase chain reaction (PCR) coupled with quantitative fluorescence detection and/or DNA sequencing techniques such as pyro-/next generation sequencing of the amplified product. Although highly sensitive and widely used, such methods can sometimes introduce errors during the PCR exponential amplification process which is sensitive to contaminations and hence may affect diagnostic accuracy.^21,22^ PCR is also often regarded as labor-intensive and time-consuming, making it unsuitable for rapid, point-of-care diagnostics. Moreover, it also requires an expensive thermal cycler and thereby the cost per analysis might be high for developing countries. These limitations have largely hampered the wide use of PCR for rapid, on-site diagnostics. In the case of point mutation detections involving miRNAs, the real-time quantitative reverse transcription polymerase chain reaction (qRT-PCR) is often the method of choice due to its high sensitivity. However, several drawbacks do exist for the qRT-PCR, including complex processes such as reverse transcription, multiple primer design, and precise temperature control.^23^ Unsurprisingly, many of the recent developments have focused on developing PCR-free alternatives in SNP biosensing. The reliable detection of low abundance specific SNPs without PCR pre-amplification represents an extremely challenging albeit exciting research area of the bioanalytical science.

Over the past few years, enormous efforts have been directed towards the development of novel, ultrasensitive PCR-free SNP assays and a number of techniques have been developed.^22,24–27^ The detection and quantification of known SNPs are primarily based on the specificity of target hybridization^28,29^ and enzyme discriminations, such as specific enzymatic cleavage,^30^ and single base primer extension.^31,32^ Of particular interest in the early stages of PCR-free SNP biosensing development has been based on using water-soluble, cationic conjugated polymers (CCPs) as fluorescence transducers. For example, the Leclerc group^33,34^ has pioneered and developed DNA detection based on the electrostatic attraction between a cationic polythiophene and DNA. The colour and fluorescence changes of the polymer in the presence of single-stranded and double-stranded DNAs were used as the basis for biosensing. They have reported the first detection of SNPs directly from clinical samples without the need of DNA pre-amplification with an impressive detection limit of 3 zeptomolar. The Bazan and Jaeger groups have also detected specific DNA sequences *via* CCP sensitised Förster resonance energy transfer (FRET) to dye-labelled probes.^35,36^ The electrostatic attraction between the cationic CCPs and the anionic DNAs leads to short distance donor (CCP)–acceptor (DNA dye-label), hence strong FRET.^37^ A substantive review of such work has already been provided by Swager *et al.* ^38^ and hence this will not be main topic here.

Recently, the use of various nanomaterials has provided the capability of ultrasensitivity and high specificity in SNP detection. Nanomaterials have been well-studied primarily because of their unique, size dependent physical and chemical properties. In terms of biosensing applications, such useful properties include extremely large surface area, tunable surface chemical composition that allow easy and controlled immobilization of stable bioreceptors for efficient transduction of target binding into strong readout signals. These properties are advantageous for achieving ultrasensitivities, allowing easy integration into miniaturized devices ushering an era of next generation cost-effective SNP diagnostics.

Nanomaterials such as metallic nanoparticles (NPs),^39–44,55^ quantum dots (QDs),^45–48^ magnetic nanoparticles (MNPs),^49–54^ and carbon based nanomaterials^56^ have been combined with biomolecules such as enzymes, oligonucleotides and DNAzymes to develop sensors for detection and quantification of cancer-specific SNPs. Such nanomaterial-based SNP assays have been coupled with a number of different readout strategies, including colorimetry, fluorimetry, surface enhanced Raman spectroscopy (SERS), electrochemical and electrochemiluminescence *etc.* For example, Boudreau and colleagues recently reported the use of FRET-based CCP transducers by combining them with highly fluorescent core–shell nanoparticles. The Ag nanoparticle core was used to amplify the optical signal generated upon target recognition. This plasmonic enhancement resulted to the direct detection of unamplified target nucleic acid at femtomolar concentration regime.^57–59^

Table 1 summarizes some of the recently developed ultrasensitive SNP assays using different nanomaterials coupled with different probe and/or signal amplification strategies. The sensitivities and specificities of these assays are already comparable to, or even surpass those of many PCR based techniques, demonstrating the high suitability of nanomaterials for ultrasensitive SNP detection. Despite significant developments, it is important to consider some other burgeoning challenges in clinical diagnosis, particularly in the quantification of extremely low abundant SNPs in an overwhelming background of wild-type genes in clinical settings. Moreover, the ability of simultaneous detection of multiple targets is also important for high diagnostic accuracy because “*no tumor marker identified to date is sufficiently sensitive or specific to be used on its own to screen for cancer*”.^60,61^ The practical importance of these relies on the fact that most cancer cases are only diagnosed in the late stage and when the chances of patient survival are already slim. As a result, early diagnosis is of paramount importance for improving the survival and prognosis of cancer patients. All these necessitate the development of ultrasensitive assays with multiplexing capability that can detect the extremely low concentrations of cancer-specific SNPs in clinically media.

**Table 1:** Comparison of the sensing performance of some ultrasensitive nano-enabled DNA detection assays^*a*^

First author ref.	Nanomaterial platform	Amplification strategy	Transducer/analytical tool	LOD
PCR based methods				
Y. Zhao^66^	Au NP + Ag NP	PCR	Circular dichroism	17 zM
F. Patolsky^84^	Magnetic NP	PCR	Chemiluminescence	8.6 aM
PCR-free methods				
W. Shen^40^	Au NP	LCR	Colorimetric	20 aM
D. Kato^75^	Au NP and magnetic NP	LCR	Colorimetric	50 zM
J. Ji^46^	CdTe QD	Target recycling + RCA	Electrochemical	11 aM
S. Bi^50^	Magnetic NP	RCA + DNAzyme	Colorimetric	71 aM
Y. P. Zeng^78^	QD	Primer generation + RCA	Fluorimetric	50.9 aM
J. W. Nam^80^	Au NP + magnetic NP	Bio-bar-code + silver amplification	Scanometric	0.5 aM
J. Hu^82^	CdS QD	CdS QD loaded in SiO_2_ particles	H_2_S generation + atomic fluorescence microscopy	0.8 aM
C. Ding^83^	Au NP + CuS NP	CuS NP loaded Au NP	Electrochemical	19 aM
R. D'Agata^85^	Au NP	Short DNA modified Au NP	Surface plasmon resonance imaging	2.6 aM
J. Zhou^86^	QD	QD entrapped in liposome	Single particle counting	1–2.5 aM

^*a*^PCR: polymerase chain reaction; LCR: ligation chain reaction; LOD: limit of detection.

In general, three different approaches are widely employed to improve the assay detection limits: target, probe, and signal amplification. Among these, target amplification is mainly achieved *via* enzyme-mediated replication of target nucleic acid sequences, leading to *ca.* 10^8^–10^9^ fold amplification of target concentration to make it high enough to be readily detected by traditional approaches such as gel electrophoresis.^22^ An excellent example here is the well established polymerase chain reaction (PCR). More recently, isothermal target amplification strategies have also been developed^24–27,62^ and displayed sensitivities comparable to that of the PCR. In contrast, probe amplification does not change the copy number of the target, but instead the probe sequence which is complementary to the target is amplified and detected by conventional hybridization method. Signal amplification strategies are approaches that amplify the analytical signal generated by each labeled or unlabelled probe so as to increase the assay sensitivity. Various signal amplification strategies have been used to increase the sensitivity of the probe-based assays.

In this mini-review, we will highlight some of the most recent advance in the field of nanomaterial-based SNP detection, with a particular focus on those techniques capable of detecting and quantifying extremely low target concentrations down to the attomolar regimes or below. Given the high specificity, the hybridization-based method remains one of the most popular methods for nanomaterial based SNP assays. In general, the ultra-sensitivity of these assays have been achieved through one of the following five different strategies, (i) nanoparticle-target assisted PCR amplification, (ii) nanoparticle-target assisted probe amplification, (iii) target recycling coupled with probe and/or signal amplification, (iv) tandem amplification coupled with signal catalytic cascades, and (v) nanomaterial enhanced signal amplification. Each of these classes will be discussed in this review with a focus on the assay sensitivity and comment on their applicability to real biological matrices. Moreover, a critical categorization of such approaches on the basis of different amplification strategies is proposed.

### 1.1 Nanoparticle-target assisted PCR amplification strategy

An essential first step in this approach is to pre-amplify the extremely low abundant SNP target into sufficiently high number of copies so that it can be readily detected by conventional methods. Given its extremely powerful exponential amplification power, it is unsurprising that PCR-based SNP biosensing assays have been reported to have high sensitivities.^63^ In particular, the incorporation of plasmonic nanomaterials such as Au and Ag nanoparticles has been recently combined with PCR pre-amplification of the target DNA, leading to unprecedented sensitivities. For example, Kotov and colleagues have reported an unexpected chirality of bridged metallic nanoparticle dimers and have exploited this in ultrasensitive biosensing. They have prepared different sized Au and Ag NPs and functionalized them with specific antibodies so that they can sandwich bind the specific target protein (*e.g.* prostate specific antigen PSA, a cancer marker) and assemble into hetero-nanoparticle dimers. These biomolecule bridged NP heterodimers are chiral, leading to an observable change in circular dichroism (CD) spectra over 350–600 nm region. The authors found that this assay was extremely sensitive, capable to detecting PSA down to the sub-aM level with an extremely impressive detection limit (DL) of 1.5 × 10^–20^ M and a linear dynamic range of ∼3 orders of magnitude. Such a highly impressive sensitivity has been attributed to plasmonic enhancement of intrinsic chirality of biomolecules, strong optical coupling of photons with twisted NP heterodimers, and bisignate nature of the CD spectra.^64,65^

**Fig. 1:**
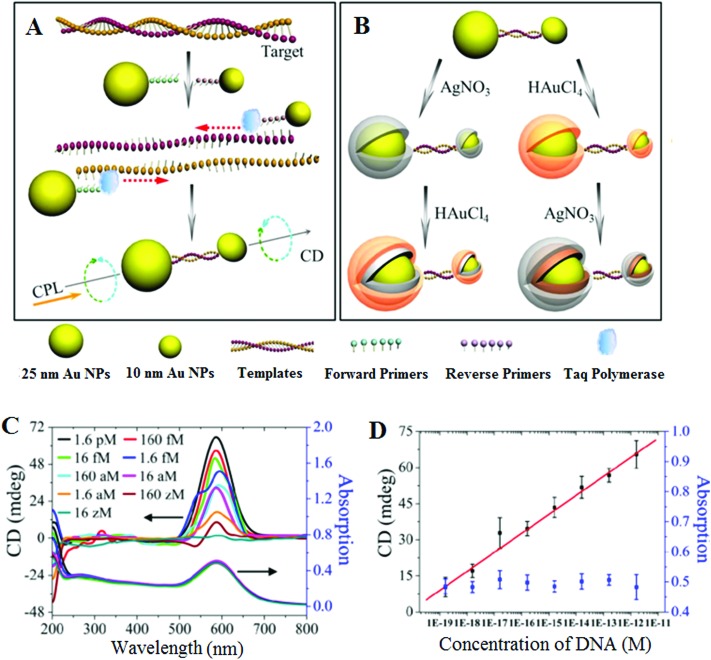
Chiroplasmonic core–shell DNA-bridged nanoparticle heterodimers. (A) Schematic illustration of the PCR based assembly of Au nanoparticle dimers. (B) Deposition of Au and Ag on the DNA-bridged Au nanoparticle dimers leading to single and multiple core–shell heterodimers. (C) CD and UV–vis spectra of Au coated heterodimers with DNA concentrations varying from 16 zM to 1.6 pM. (D) Calibration curve relating the intensity of CD bands of Au coated heterodimers and the concentration of DNA. Reprinted with permission from ref. 66, copyright©2014, American Chemical Society.

This assay has been recently modified to suit the detection of ultralow concentrations of DNA^66^ by applying a PCR amplification of the dsDNA with primers linked to Au NPs (25 nm Au NP-linked forward primer and 10 nm Au NP-linked reverse primer) in the presence of Taq polymerase and dNTPs (see Fig. 1). The DNA-bridged Au NP heterodimers displayed circular dichroism (CD) bands at 260 and 525 nm, attributed to the chiral secondary structures of duplex DNA and the chiral NP dimers, respectively. These dimers displayed unexpected chirality due to the ellipsoidal shape of the NPs and a scissor-like configuration with two long NP axes forming a dihedral angle of 10°. Coating of the Au NP dimers with Ag or Au are found to affect their chiro-plasmonic activity: as the thickness of the Ag shell increased, the intensity of the CD bands also increased and shifted from 525 to 418 nm. In the case of Au coating, increasing the Au shell thickness resulted in a color change of the dispersion from pink to purple and the CD bands exhibited a 61 nm red shift. More importantly, Au coating of DNA-bridged Au NP dimers gave fairly narrow spectra with high optical anisotropy. Optimization of assay parameters such as specific design of primers and number of PCR cycles has yielded ultrasensitive DNA assay with a linear dynamic range spanning 7 orders of magnitude (from 160 zM to 1.6 pM) and an extremely low DL of 17 zM. Currently, this method reported the highest sensitivity for DNA detection using nanomaterials based sensing platforms, and hence appears to have good potential for genetic based early diagnostic applications. Despite such great sensitivity, the applicability of this assay towards the detection and discrimination of specific SNP's in complex media such as the wild-type DNA background has yet to be demonstrated. Moreover, given the fact that this assay requires PCR pre-amplification, it is less well-suited to applications that require rapid results such as the point-of-care diagnostics.

### 1.2 Nanoparticle-target assisted probe amplification strategy

Rolling circle amplification (RCA)^67–69^ and ligase chain reaction (LCR)^70–72^ are two of the most extensively used probe amplification strategies in SNP detections. Recently, nanomaterials have been combined with these probe amplification assays, leading to further improvements in detection limits. These assays typically use a complementary oligonucleotide capture probe chemically linked to an Au nanoparticle, quantum dot, or magnetic nanoparticle to directly interact with their specific analytes, thereby amplifying molecular recognition events such as DNA hybridization. In addition, magnetic nanoparticles have been further used for target capture, enrichment and purification which can further enhance the assay sensitivity.

**Fig. 2:**
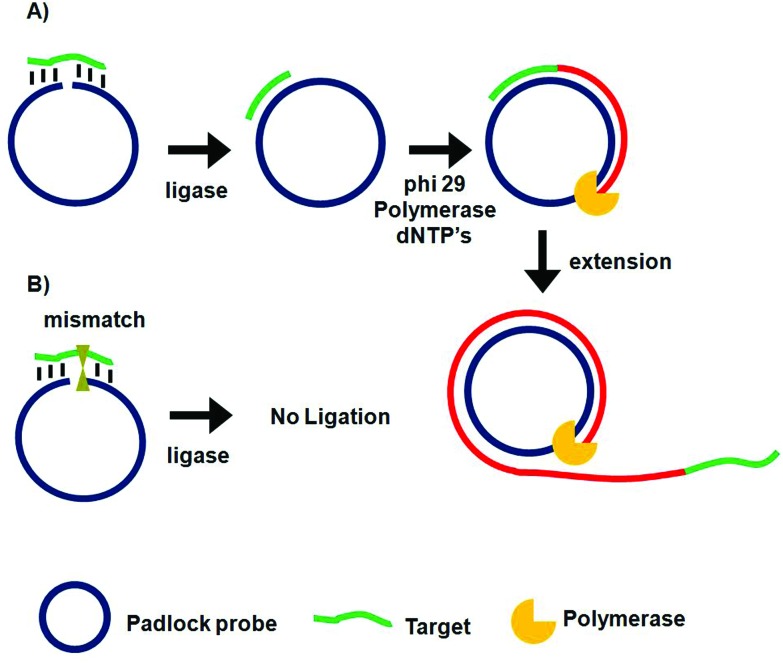
Schematic principle of the rolling cycle amplification and SNP discrimination. (A) Hybridization of the perfect-match target DNA to circular padlock probe leads to covalent ligation, producing a target DNA hybridized circular probe where in the presence of phi29 polymerase and dNTP's, the target serves as primer to initiate the circular extension of long single-stranded DNA with repeat sequence complementary to the padlock probe. (B) The presence of a single base mismatch between the target DNA to the padlock probe leads to no ligation and hence no amplification.

Unlike PCR which requires thermal cycles, RCA is an isothermal probe amplification strategy using a single-stranded DNA as a padlock probe (Fig. 2A). The 5′-(phosphate modified) and 3′-terminal fragments of the padlock probe are specifically designed to be complementary to the target DNA. Hybridisation of the target to the padlock probe then brings its 5′- and 3′-terminus close to each other, forming a nicked circle which can be covalently ligated together by a DNA ligase if the padlock and target sequences are fully-matched, forming a circularized padlock probe. The circularised padlock probe is then amplified by a φ29 polymerase in the presence of dNTP's, producing a greatly elongated single-stranded DNA containing numerous copies of repeat tandem sequence units complementary to the circular padlock probe. In contrast, the presence of a single-base mismatch at the nicked target/padlock duplex probe can prevent the specific ligation and formation of the circular padlock probe, and therefore no RCA amplification (Fig. 2B). The stringent requirement of full sequence complementary between the padlock and target to form the ligated circular padlock probe allows the RCA strategy to have excellent specificity for SNP detection.^42,77^

**Fig. 3:**
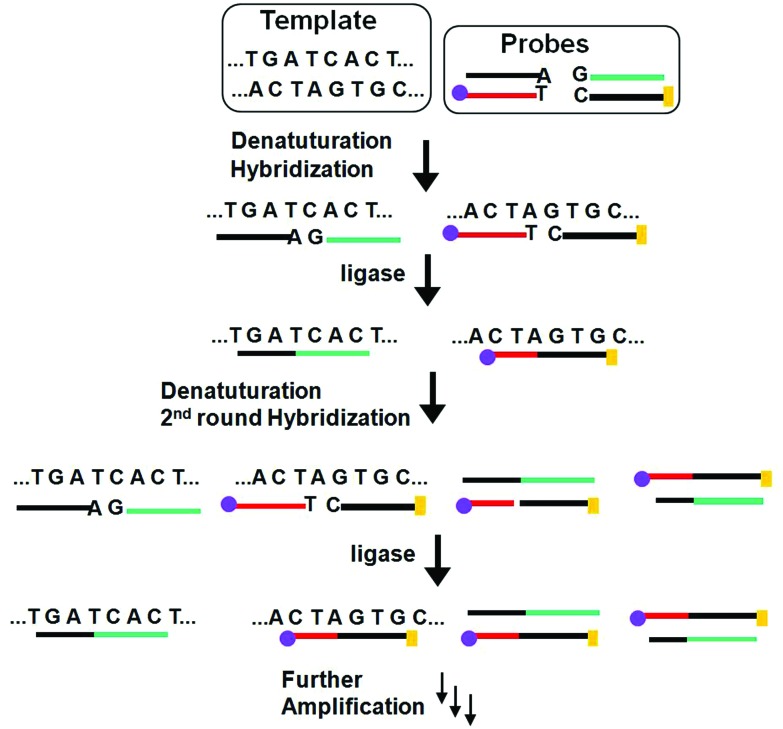
Schematic principle of the ligase chain reaction (LCR) based DNA amplification strategy. Each of the two DNA strands in the duplex target serves as a template to ligate its respective two short DNA strands, leading to doubling of the ligated product in each cycle and hence an exponential amplification of the target DNA. The amplified target sequences can then be detected by using their specific 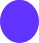 and 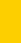 tags.

Similar to PCR, the ligase chain reaction (LCR) also requires multiple thermal cycles to achieve specific ligation of two short DNA probes catalysed by a DNA ligase into a single strand templated by a full-match DNA target.^24–27^ In a typical SNP assay, LCR uses two pairs of probes each containing two short complementary oligonucleotides, but the overall sequence is complementary to the target DNA sequence (Fig. 3). After thermal denaturation followed by annealing, a pair of the probes are hybridized to one of the denatured target DNA strand. They are subsequently ligated by a DNA ligase if the probes and target sequences are fully complementary at the nick site. On the other hand, the presence of single-base mismatch at the nick site can prevent specific ligation, allowing for the LCR to have high SNP selectivity. After the first ligation round, each ligated product can then function as a new template to ligate further probes in the following thermal cycles. Repeating the thermal cycles thus leads to an exponential increase of the ligated probes. Ligation reactions have better single-base mismatch discriminatory ability than primer extension methods, making LCR more specific for SNP detection than PCR based methods.^24^

A particularly attractive SNP assay has been the colorimetric sensing using Au NPs because of their extremely strong surface plasmon resonance (SPR) absorption at ∼520 nm (*ε* > 10^9^ M^–1^ cm^–1^ for a 20 nm Au NP, 4–5 orders of magnitudes stronger than typical organic dyes), making it strongly colored even at low nM concentrations. Moreover, its SPR band is also sensitive to aggregation, isolated Au NPs appear red but aggregated ones are blue or purple. The resulting color change is distinct and clearly visible by the naked eye.^27^ Here specific DNA targets that can induce the aggregation of Au NPs have been used to detect SNPs, taking the full advantage of the very sharp melting transition for the sandwich duplex formed between the target DNA and a pair of probe DNA (with sequence complementary to each half of the DNA target) modified Au NPs. The sharp melting transition means a single-base mismatch between the target and probes can be distinguished due to their slightly different thermal stability. This leads to distinct color changes at elevated temperatures that can be exploited for SNP detection.^73,74^ Combined with various amplification strategies, such assays have achieved pretty low detection limits, down to the femtomolar region.

**Fig. 4:**
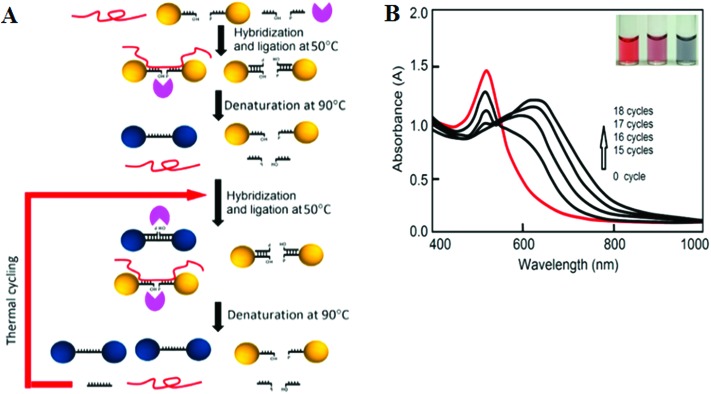
(A) Schematic illustration of the real-time Au NP mediated LCR assay. (B) UV–Vis spectra of the solution containing 100 fM target DNA and 10 nM CP-coated AuNPs during the LCR process. Inset: color of solution after addition of 0, 5, and 50 nM (left to right) target DNA to 10 nM CP-coated AuNPs. Reprinted with permission from ref. 40, copyright©2012, American Chemical Society.

More recently, LCR has been combined with Au NP for ultrasensitive colorimetric SNP detection by the Gao group.^40^ The assay involves the real time ligation of oligonucleotide coated Au NPs templated by the complementary SNP target (Fig. 4A). In each LCR cycle, there is an increasing amount of Au NPs being ligated that are subsequently used to template the ligation of further Au NPs, leading to an exponential increase in the amount of covalently linked Au NPs. Since the SPR band of Au NPs is sensitive to NP assembly, this resulted in a color change that can be directly monitored by UV-vis (Fig. 4B). This assay has an impressive linear dynamic range of 6 orders of magnitude and is capable of detecting specific DNA targets down to 20 aM. This assay also has high selectivity: it can specifically detect the wild-type (WT) KRAS gene in the presence of large excesses of genomic DNAs even at 1 : 100 000 (KRAS : genomic DNA) base pair ratios when readings were taken at 90 °C. At this temperature, all non-chemically ligated DNA–Au NP conjugates were dislodged, leaving only the covalently ligated Au NPs in the solution. Thus, the incorporation of ligation allowed for easy elimination of interferences from coexisting DNA and a reduced background. This assay has been further detected the WT-KRAS in the presence of KRAS single point mutants, yielding an impressive SNP selectivity factor of 2000. This assay can be used as an efficient approach for detecting specific mutant DNAs by simply redesigning the sequences of capture probes and signal probes, and thus has great potential for ultrasensitive detection of various disease-related SNPs. Despite such great promises, the relatively long assay time and requirement of multiple thermal cycles may limit its use in rapid, point-of-care applications.

**Fig. 5:**
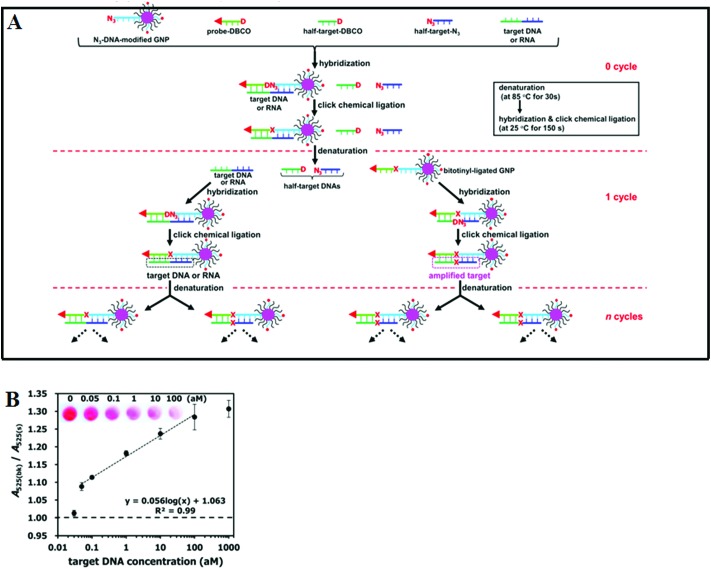
(A) Schematic illustration of Enzyme Free Click Chemical Ligation on Au nanoparticles involves the hybridization of the target DNA with an azide-modified Au NP (N_3_-AuNP), dibenzocyclooctyne-modified probe (DNCO-probe). (B) A plot of the A_525_(bk)/A_525_(s) ratio at different target DNA concentrations using 40 thermal cycles. Inset shows a photograph of the supernatants at different target DNA concentrations. Reprinted permission from ref. 75, copyright©2014, American Chemical Society.

Although the LCR strategy appears to be a highly attractive alternative to PCR amplification, an apparent weakness has been its low amplification efficiency when being combined with nanomaterials. This is due to the restricted accessibility of the ligases to the ligation site in oligonucleotide modified Au NPs. In an attempt to overcome the limitations of conventional LCR, a process called enzyme-free click chemical ligation reaction (CCLR) that involves Au NP and magnetic bead was developed by Kato and colleagues^75^ (Fig. 5A). Unlike the earlier cross-linked Au NP systems, this assay does not require the use of ligases to carry out LCR and does not require the formation of large assemblies. The CCLR method uses an azide-containing DNA modified Au NP and a dibenzocyclooctyne-containing biotin-DNA probe. Sandwich hybridization of the target DNA (RNA) with the azide-DNA modified Au NP and biotin-DNA lead to enzyme-free ligation *via* the copper-free click chemistry, producing biotin-ligated Au NPs. Repeating the thermal cycles lead to the biotin-ligated Au NPs being exponentially amplified, which are then captured by using streptavidin-modified magnetic beads. After magnetic separation, the strong absorption of the Au NP can be used to detect and quantify the target DNA (RNA). Using this assay strategy, a DNA sequence associated with the hepatitis A virus Vall 7 polyprotein gene (HAV) has been detected at a concentration as low as 50 zM with a linear dynamic range of 3 orders of magnitude (Fig. 5B), which is highly impressive. However, it should be noted that the absolute signal difference throughout the whole dynamic range was relatively small, being only ∼30% (*e.g.* increased from ∼1.0 to 1.3), which can significantly limit its diagnosis accuracy. This method can also discriminate specific single point mutations, with the G-, T-, and C-Mutant signals being 17, 0, and 0% of that of the full-match control respectively. This shows that this essay has an excellent SNP discrimination, although a more useful demonstration of the SNP specificity would be the ability to detect low specific target SNPs in a background of wide-type genes under clinical relevant media.^75^

### 1.3 Target recycling coupled with probe and/or signal amplification

Target recycling is another useful strategy to amplify low copy number SNPs. In this strategy, the target DNA is cycled in a number of hybridization events, each time producing one or several copies of the complementary probe strand, depending on the approaches used. However, the linear amplification nature of target recycling process has largely limited its detection limit to the fM regime only.^53,76^

**Fig. 6:**
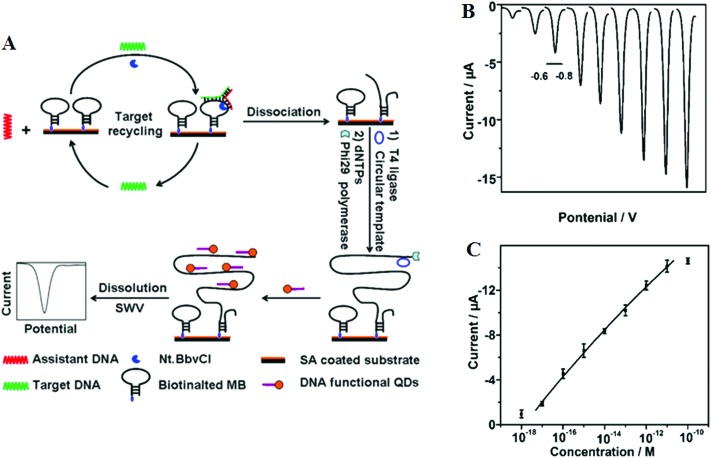
(A) Schematic illustration of the TEHP amplification strategy for DNA detection, (B) stripping voltammetric curves of cadmium ions corresponding to (left to right) 10^–18^, 10^–17^, 10^–16^, 10^–15^, 10^–14^, 10^–13^, 10^–12^, 10^–11^, and 10^–10^ M of target DNA. (C) The corresponding analytical quantitative dynamic range. Reprinted permission from ref. 46, copyright©2012, American Chemical Society.

More recently, target recycling has been combined with several other probe amplification strategies to further improve detection limits. A significantly higher sensitivity has been achieved by Ju and colleagues^46^ who have designed a *template enhanced hybridization process or TEHP* coupled with rolling cycle amplification. This target recycling hybridization assay uses a biotinylated molecular beacon [MB] bound to a streptavidin coated plate as the template. Hybridization of the target DNA and an assistant DNA to the loop region of the MB forms a “Y” shaped junction (Fig. 6A). This configuration provided a specific nucleotide sequence that can be nicked by a suitable endonuclease. Once released, the target DNA and assistant DNA initiate another round of hybridization and strand scission cycle. The numerous MB fragments left in the plate then served as primers for the RCA process to produce thousands of repeat oligonucleotide sequences. These repeated sequences then are hybridized to an oligonucleotide functionalized CdTe QD and separated. The QDs are then dissolved by an acid, releasing greatly amplified Cd^2+^ ions that are detected by square wave voltammetry. The combination of TEHP and RCA coupled with the use of QD based signal tags has offered an enormous amplification of the DNA target, allowing for greatly increased assay sensitivity. An impressive sensitivity of 11 aM has been attained together with a wide dynamic range of 6 orders of magnitude (Fig. 6B and C). This assay was also able to discriminate the perfect complementary DNA target against various mismatch targets with a discrimination ratio of 3.8, 5.7 and 6.6 fold for the single-base, three-base mismatch and non-complementary oligonucleotides, respectively. Despite a high sensitivity, the mutant discrimination ratio here is relatively moderate compared to other specific SNP assays, which may limit its potential for applications in real clinical samples where the presence of large background genomic DNAs and/or wild-type genes may strongly interfere the specific SNP detection.

### 1.4 Tandem amplification schemes and signal catalytic cascades

In order to achieve detections in even lower concentration regimes, several researchers have reported the use of tandem amplification schemes.^42,77,78^ The combination of two amplification strategies has been deemed necessary to further improve the sensitivity and specificity of SNP detection. Moreover, this strategy can overcome the low amplification efficiency by a single amplification scheme.

The colorimetric detection of SNPs using a combination of RCA and nicking endonuclease-assisted nanoparticle amplification strategy was developed by Xu and colleagues.^42^ In this assay, ligation was performed when the target DNA was hybridized with the padlock probe, leading to a circularized template. Subsequent RCA reaction in the presence of dNTPs led to the formation of a long single strand DNA. Nicking reactions at many of the repeated sites along the RCA elongated single-stranded DNA occurred simultaneously. Upon completion of the strand scission cycles, the addition of a specific oligonucleotide modified Au NP provided a simple, colorimetric detection of target DNA down to 1 pM.

The sensitivity of ligation assays can be further improved when being coupled to another probe amplification scheme. For example, the combination of LCR and RCA in detecting SNPs has been recently demonstrated by Cheng and colleagues,^77^ who has demonstrated the sensitive detection of 1 fM of unlabeled target DNA under the optimised assay conditions.

**Fig. 7:**
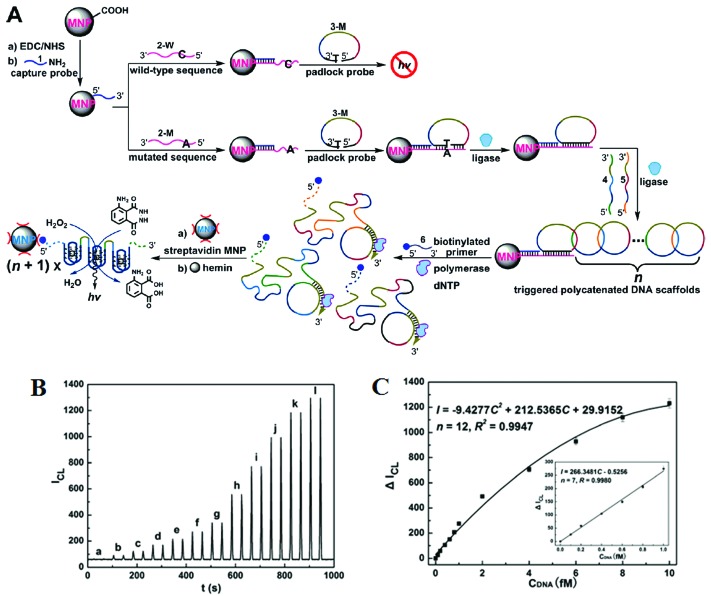
(A) Schematic representation of interlocked DNA scaffold mediated RCA reaction and DNAzyme amplification assay for the detection of G12C mutation in the KRAS gene. (B) Chemiluminescence signals for the HRP-mimicking DNAzyme-catalyzed luminol-H_2_O_2_ system corresponding to different concentrations of single-base mutant target DNA: (a) 0; (b) 0.1; (c) 0.2; (d) 0.4; (e) 0.6; (f) 0.8; (g) 1.0; (h) 2.0; (i) 4.0; (j) 6.0; (k) 8.0; (l) 10.0 fM. (C) The corresponding calibration curve of peak height *versus* the concentration of target DNA. Reprinted with permission from ref. 50, copyright©2010, American Chemical Society.

Further improvements of assay sensitivity have been achieved by incorporation of DNAzymes (catalytic nucleic acids) based catalytic cascades signal amplification. In this regard, Zhang and colleagues^50^ have designed a new RCA amplification approach where the presence of the target KRAS SNP triggers the ligation of the padlock probe. Subsequently, multiple other circular templates were interlocked to the padlock probe by means of complementary sequence forming an ABABAB-type DNA copolymer (Fig. 7A). The inter-locked circular primers containing the HRP-mimicking DNAzymes subsequently underwent another round of RCA, producing long, single-strand DNA products each containing thousands of copies of the repeated DNAzyme sequences acting catalytic units. The incorporation of numerous such catalytic units thereby greatly enhanced the chemiluminescence signal in the presence of luminol and H_2_O_2_, leading to an impressive detection limit of 71 aM for the target SNP. This assay also provided excellent performance in quantitative analysis in human blood serum with a linear dynamic range of 2 orders of magnitude with good signal linearity from 0.1–10 fM (Fig. 7B and C). The excellent sensitivity of this assay lies on the inherent capacity to generate great amplification as a consequence of the high turnover reaction of DNAzymes. This assay was also highly specific, and can detect 1 fM specific mutant target in the presence of 10 pM wild-type gene background. It demonstrated a SNP detection capability of 1 in 10 000 (SNP *versus* wild-type target) level, which is among the best reported in literature. Hence, combining high sensitivity and specificity, this assay appears to have excellent potential for SNP based disease diagnostics by specific detection of the low abundant mutated target in clinical laboratory settings. This assay, however, is relatively complex and requires multiple amplification cycles, making it less well-suited for rapid, point-of-care applications.

Recently, the Zhang group has developed an ultrasensitive miRNA assay based on primer generation-mediated rolling circle amplification [PG-RCA]. This assay was used to analyze the point mutation of mir-196a2 [T→C] in the lung tissue samples of non small cell lung cancer patients.^78^ In this assay, the presence of target mir-196a2T circularizes the padlock probe and the mir-196a2T further functioned as a primer to initiate the next round of RCA reaction in the presence of Vent (*exo*-) polymerase. This resulted in a long single-stranded RCA product containing numerous restriction sites for *Nb.BsmI*. The RCA product was then nicked by *Nb.BsmI*, generating a large number of new primers that was further used to initiate a new RCA reaction. The amplified DNA products were subsequently hybridized with a biotin/Cy5-labeled capture probes to form a double-stranded DNA. This DNA duplex contains a recognition site for the *Nt.BstNBI* nicking enzyme, and after the nicking reaction the capture probe was cleaved, separating Cy5 and biotin. This also resulted in the release of the amplified DNA product wherein it can repeatedly hybridize with new biotin/Cy5-labeled to initiate the next rounds of cleavage. In a similar strategy, mir-196a2C was detected using the designed mir-196a2C padlock probes. In the absence of the mir-96a2T, more Cy5/biotin capture probes are cleaved, leading to smaller amounts of the intact capture probes that can bind on the surface of the streptavidin-coated QD. Consequently, this reduces the FRET between the QD donor and Cy5 acceptors, resulting in decreased Cy5 counts being detected in the single-particle FRET detection measurement. On the other hand, the mir-196a2T-specific linear padlock probe cannot be circularized in the absence of mir-196a2T. Under these conditions, RCA amplification and cleaving reactions will happen. Hence, the biotin/Cy5-labeled capture probe remains intact which can bind to the QD surface *via* specific streptavidin–biotin interactions, resulting in strong QD sensitised Cy5 FRET signal. With the integration of the PG-RCA reaction and nicking enzyme-driven recycling amplification, this QD-based miRNA nanosensor exhibited an impressive detection limit of 50.9 aM and a large dynamic range of 7 orders of magnitude from 0.1 fM to 1 nM for the specific microRNA target. Moreover, this assay can even distinguish variant frequencies down to as low as 0.001% in the mixtures of mir-196a2C and mir196a2T. Importantly, this QD-based miRNA nanosensor can be used to analyse the point mutation of mir-196a2 in the lung tissues of NSCLC patients, holding great potentials for further applications in biomedical research and clinical diagnosis presumably in the clinical laboratory settings. Its complex signal amplification and assay procedures together with a relatively complex single-particle counting readout method may, however, limit its use in applications that require rapid results.

### 1.5 Nanomaterial assisted signal amplification

In signal amplification, nanomaterials are often functionalised with biomolecules for target-specific recognitions and/or carrying high loads of signal moieties, catalysts, optical emitters, and electronic conductors. Biofunctionalised nanomaterials can amplify the signal transduction events due to their capability of direct interaction with their target, allowing for detection of biomolecules down to the single-molecule level.^79^ Signal amplification can eliminate some special requirements of the target/probe amplification schemes such as the need of enzymes and thermal cycles (*ca.* PCR). As a result, this can simplify experimental protocols, lower the assay cost, and provide amenability to miniaturisation. Hence, the use of biofunctionalized nanomaterials for signal amplification has been very attractive in the development of ultrasensitive DNA assays without the need of target and probe amplifications. The following section presents some ultrasensitive detection of SNPs achieved through use nanomaterials for signal amplification.

#### 1.5.1 Nanoparticle bio-barcode amplification

**Fig. 8:**
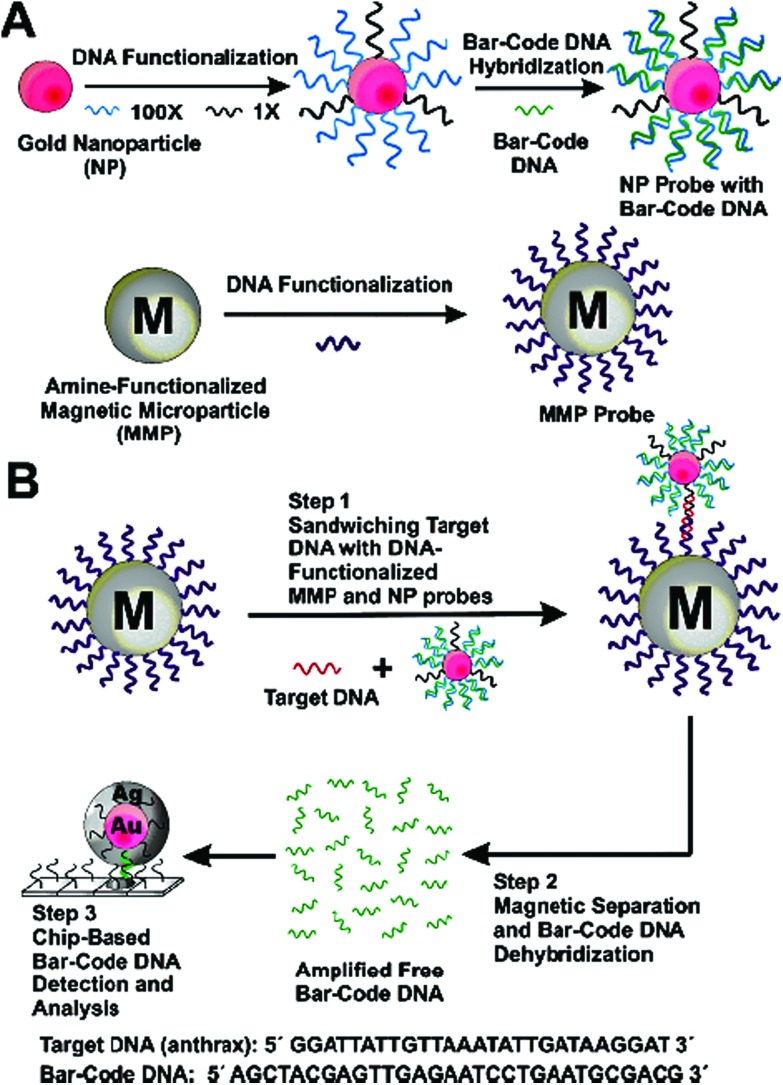
Schematic illustration of the DNA-Bio-bar-code based ultrasensitive assay for DNA detection. (A) The preparation of the DNA modified Au NP and magnetic microparticle probes. (B) Schematics of the nanoparticle-based PCR-free DNA amplification and detection scheme. Reprinted with permission from ref. 80, copyright©2004, American Chemical Society.

An excellent example of the earlier developments here is the Bio-barcode assay developed by the Mirkin group.^80^ It uses a short DNA-modified An NP tagged with hundreds of copies of the bio barcode DNAs (Bbc-Au NP) as signal probe, and another DNA-modified magnetic microparticle (Oligo-MMP) as capture probe (Fig. 8). The two DNAs are complementary to each half of the target DNA, so that they can sandwich bind to the target DNA, forming a Bbc-Au NP/DNA target/Oligo-MMP hybrid structure. After magnetic separation and washing to remove any unbound species, thermal denaturation of the hybrid releases the bio barcode DNAs, converting each capture DNA target into hundreds of copies of barcode DNAs. These are then detected by a sensitive scanometric assay coupled with silver amplification to achieve ultrasensitivity.^81^ This assay provides an impressive label-free detection of target DNA down to 500 zM, comparable to many PCR based methods.^80^ It can also discriminate the perfect match DNA from the SNP target. Although its relatively modest discrimination ratio, ∼3 : 1, may limit its capability of detecting low abundant SNP targets in wild-type gene background.

#### 1.5.2 Signal amplification using labeled and enriched nanoparticle probes

Metallic nanoparticles can be employed as scaffold for loading not only the capture but also signal molecules. These labelled nanoparticles present an important signal amplification strategy as they can directly enhance the readout signal for each target and probe recognition while retaining a high binding specificity. Over the past few years, silica particles have been used as another carrier platform due to their large surface areas, well-known surface chemistries and good biocompatibility.

Wu and colleagues have reported a labeling-based signal amplification strategy for ultrasensitive detection of target DNA using QD assembled SiO_2_ microspheres.^82^ This method is used as an indirect method for ultrasensitive detection of HIV DNA using hydride generation atomic fluorescence spectrophotometry [HG-AFS]. This assay involves the use of sandwich hybridization between the target DNA, biotin-capture probe and streptavidin tagged QD-SiO_2_ assembly, converting each captured DNA target into thousands of SiO_2_ tagged QDs. After acid dissolution of the QDs, the resulting Cd^2+^ ions were detected by HG-AFS, giving a highly impressive detection limit of 0.8 aM for the target DNA and a dynamic range of 3 orders of magnitude (from 1 aM to 1 fM). The outstanding sensitivity suggests that the HG-AFS method is suitable for the ultrasensitive biosensing.

An ultrasensitive chemiluminescent method using Au NP based signal amplification has recently been developed by Zhang and colleagues.^83^ Using the Watson–Crick base pairing, a guanine monomer modified Au NP probe is coupled to the cytosine mutated DNA duplex in the presence of DNA polymerase I. Each guanine modified Au NP probe is also linked to 77 CuS NPs which act as signal generator. After acid dissolution of the CuS NPs, the released Cu^2+^ ions are coordinated to cyanides to form [Cu(CN)_4_]^2–^ complexes. These are then reacted with luminol to give rise to chemiluminescence signal for target DNA quantification. A further improvement in the signal sensitivity is achieved by incorporating a pre-concentration of Cu^2+^ ions by anodic stripping voltammetry (ASV). These have enabled this assay to achieve a low detection limit of 19 aM for one base mutant DNA and a linear range of 80 aM to 10 fM.

The labeling of the target DNA with a large number of signal generators coupled with sensitive electrochemiluminescence detection is very powerful towards ultrasensitivity for SNP detection as demonstrated by the Willner group.^84^ In this assay, a mutant DNA with one base mismatch (C→G mutation) is first hybridised to a complementary DNA modified magnetic NP. It is then treated with a DNA polymerase in the presence of biotin-dCTP and other dNTPs. This is then followed by multiple thermal cycles of dissociation, annealing and labeling, resulting in poly-labeling of the magnetic NPs with biotins. After magnetic separation and treatment with avidin-horseradish peroxidase (Avidin-HRP), the resulting avidin-HRP functionalised magnetic NPs and napthoquinone NPs are deposited on an electrode guided by an external magnetic field. Applying an electric potential reduces napthoquinone to hydroquinone, which simultaneously reduces O_2_ to H_2_O_2_, triggering the HRP catalysed oxidation of luminol and yielding a chemiluminescence signal. Under optimized conditions, this assay has reported an impressive sensitivity of 8.3 aM for the M13φ DNA (50 copies in 10 μL sample) and 10 aM for a mutant DNA.

**Fig. 9:**
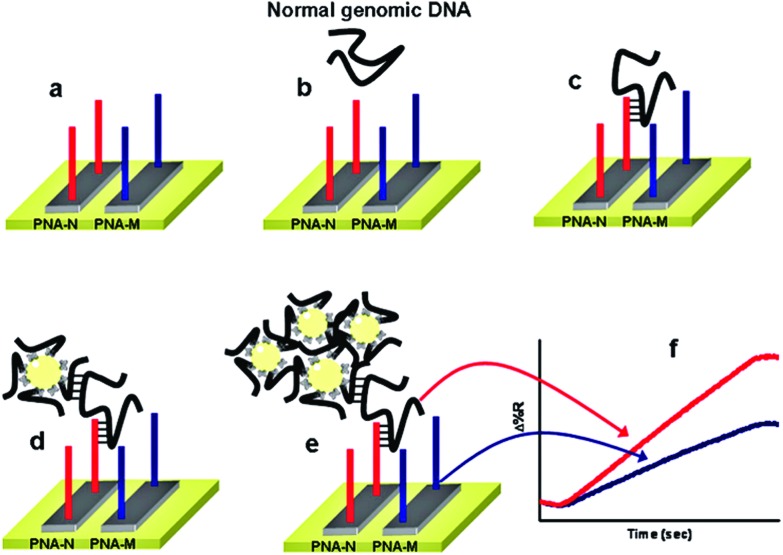
Schematic illustration of the nanoparticle-enhanced SPRI strategy used to detect the normal βN/βN, heterozygous β°39/β°N, and homozygous β°39/β°39 genomic DNAs. The PNA-N and PNA-M recognize specifically the normal β-globin and the mutated β°39-globin genomic sequences, respectively. Reprinted with permission from ref. 85, copyright©2011, American Chemical Society.

Despite showing impressive sensitivities, none of the above ultrasensitive assays have reported the simultaneous detection of multiple targets. Further developments on the multiplexing capability of an SNP assay are highly desirable for improving the diagnostic accuracy. Recently, Gambari and colleagues^85^ have reported the direct detection of SNPs in non-amplified human genomic DNA carrying the mutated β°39-globin gene sequence by using surface plasmon resonance imaging (SPRI). This gene is involved in the hereditary blood disorder diseases known as β-thalassemia. The detection is achieved by using Au NP conjugated with multiple copies of DNA β°39, an 11-mer oligonucleotide. Prior to analysis, the surfaces of 6 microfluidic channels are modified with PNA-N and PNA-M probes whose nucleic acid sequences are complementary to the normal and mutant genes respectively (Fig. 9). The samples are directly fluxed into these channels to allow the direct hybridisation between each of the samples (*e.g.* normal βN/βN; homozygous β°39/β°39 and heterozygous β°39/βN) on PNA functionalized surfaces. The SPRI responses between the samples and the two different PNA probes are used as controls. Subsequently, the conjugated Au NPs are fluxed into the microchannels and captured by specific hybridization between their surface DNA β°39 strands and exposed target DNA sequence not involved in binding to the PNA probes, allowing for greatly enhanced SPRI signal. This assay has achieved the sensitive detection of genomic DNAs down to 2.6 aM (5 pg μL^–1^) without PCR amplification. It can also discriminate between the normal, homozygous β°39/β°39, and heterozygous β°39/βN sequences, albeit with a relatively modest discrimination ratio.

#### 1.5.3 Signal amplification using functionalized nanocontainers

The use of nanomaterials with hollow structures for encapsulation of signaling elements is another attractive strategy for ultrasensitive biosensing. Lin and colleagues^48^ have used a similar base-pairing mechanism to target DNA point mutations using a novel signal amplification strategy. It uses DNA polymerase I (Klenow fragment) to couple a guanine-modified NP probe to the mutation site of a duplex DNA. The signal enhancement is achieved by incorporating Cd^2+^ ions inside an engineered protein with a hollow cage of interior cavity diameter of 8 nm. This NP probe is also modified with guanine nucleotide to specifically pair with cytosine point mutation in the DNA duplex. As a result, hybridization of the mutant DNA (cytosine point mutation) and the biotin-labeled DNA probe forms a stable duplex DNA structure which is then captured by avidin-modified magnetic NPs. The Cd^2+^ ions released from the nanoparticle cavity is then detected electrochemically by square wave voltammetry. This assay is sensitive, allowing for detection of 21.5 attomol mutant DNA and can detect SNPs down to frequencies as low as 0.01.

**Fig. 10:**
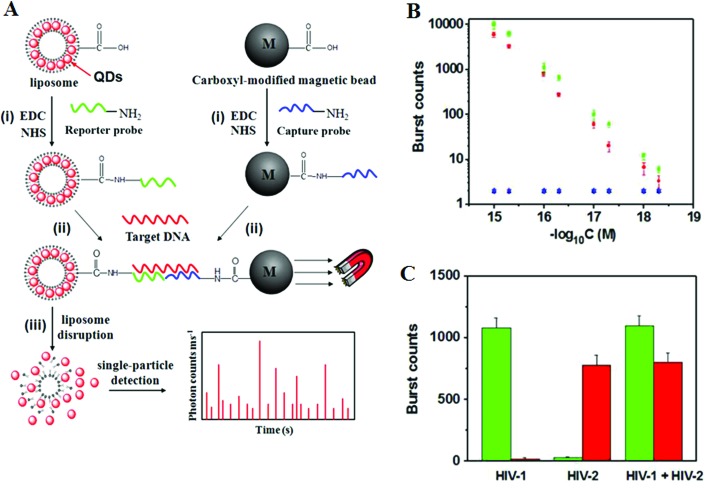
(A) Schematic illustration for Liposome–QD complexes (L@QD complex) based ultrasensitive detection of attomolar DNA using single-particle detection techniques. The assay involves the sandwich type hybridization of the target DNA to L@QD complex-tagged reporter probes and capture probe modified-magnetic bead. Liposome disruption leads to the release of QD's for single particle detection. (B) A plot of burst counts from the released QDs as a function of the concentrations of HIV-1 (green) and HIV-2 (red). There was no change in the burst counts in the control groups with non-complementary DNA (black and blue), and (C) Simultaneous detection of HIV-1 (green) and HIV-2 (red). The error bars corresponds to standard deviation of three replicates. Reprinted with permission from ref. 86, copyright©2013, American Chemical Society.

Recently, Zhang and colleagues^86^ has developed a new approach using QD-based SNP detection without the need for target or probe amplification. This assay uses a reporter probe modified-liposome (liposome–QD-reporter probe; L@QD complex) serving as a “nano-container” for encapsulation of hundreds of QDs. The presence of the target DNA (T-DNA) and capture probe modified magnetic bead (CP-MB) lead to sandwich hybridization, forming a liposome–QD-reporter probe/T-DNA/CP-MB hybrid structure which is then separated magnetically. The subsequent disruption of liposome–QD complexes results in release of the encapsulated QDs, which are sensitively detected by single-particle counting (Fig. 10). An advantage of this approach is that each pair of reporter/capture probes can be encoded with a different coloured QD, allowing for easy detection of multiple DNA targets simultaneously.

As shown in Fig. 10, the presence of HIV-1 only produces fluorescence bursts from the green QDs but not the red QDs. In contrasts, the presence of HIV-2 only produces red QD fluorescence, but not green QD, and thus indicating high assay specificity. Multiplexing has been achieved by encapsulating red and green QDs into the respective reporter probe 1 and reporter probe 2 tagged liposomes, whereupon specific sandwich hybridization lead to the formation of specific magnetic bead-liposome complexes. After magnetic separation, the use of two different coloured liposome–QD complexes has enabled the simultaneous detection of the HIV-1 and HIV-2 genes (Fig. 10B and C). The great target amplification (each captured DNA targeted is converted into several hundreds of QDs) coupled with sensitive single-particle counting method has enabled this assay to be ultrasensitive, with detection limits down to 1 and 2.5 aM for HIV-1 and for HIV-2, respectively. With the judicious design of capture and signal probes, this assay may provide an ultrasensitive approach for simultaneous detection of multiple DNA targets. However, this assay is unlikely to be able to provide high enough SNP discrimination ratio useful for detection of specific low abundant SNPs in wild-type gene background because of the small stability difference of the sandwich DNAs for the wild-type and SNP DNAs formed with the reporter/capture probes. Its single-particle counting readout strategy may also limit its application in resources poor environment.

#### 1.5.4 Signal amplifications using enzyme guided crystal growth

**Fig. 11:**
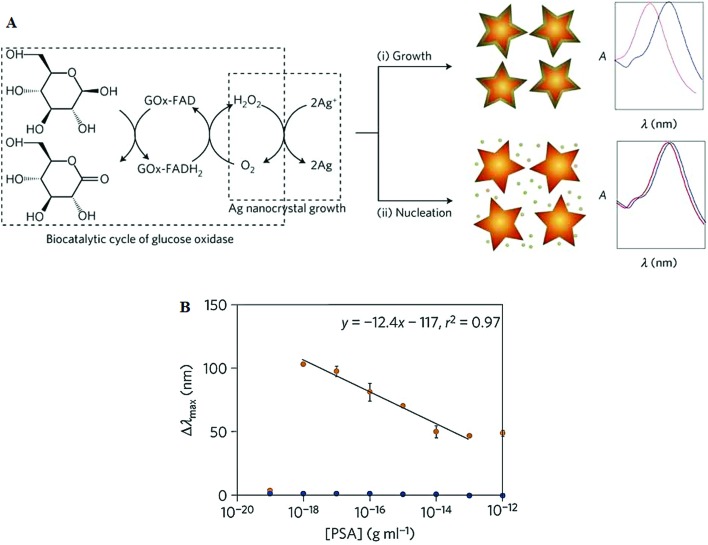
(A) Schematic illustration of the proposed signal-generation mechanism by means of enzyme-guided crystal growth for the plasmonic ELISA assay. (B) Immunoassay for the ultrasensitive detection of PSA with GOx-labelled antibodies and of PSA (red) and BSA (green) spiked into whole serum. Reprinted with permission from ref. 87, copyright©2012, Nature Publishing group.

Stevens and colleagues have recently developed a novel plasmonic nanosensor that works on a principle of inverse sensitivity signal-generation mechanism,^87,88^ where an amplified signal is observed when less target molecules are present. The inverse sensitivity procedure is achieved using glucose oxidase (GOx) which catalyses the generation of hydrogen peroxide to tailor the plasmonic response of the gold nanosensors. Depending on the concentration, GOx can control the *in situ* rate of nucleation of nascent Ag nanocrystals or Ag coating on Au nanostars, resulting in variations of the localized surface plasmon resonance (LSPR). At low GOx concentration, a low supply of H_2_O_2_ favours the deposition of a homogeneous silver coating on the Au nanostars, leading to blueshift of the LSPR band. Whereas a high GOx concentration favours the nucleation of silver nanocrystals instead of depositing on Au nanostars, leading to less LSPR shifts (Fig. 11). This principle is used to develop a plasmonic ELISA using a GOx conjugated antibody in a typical sandwich immune-binding format. The concentration of GOx is directly related to the target concentration. Prostate specific antigen (PSA) and HIV-1 capsid antigen p24 are used as model protein targets in whole serum conditions. This assay has demonstrated an extremely impressive sensitivity of 40 zM^87^ and 1 aM^88^ for PSA and HIV-1 capsid antigen, respectively. Moreover, the assay results can be directly visualised by the naked eye, and therefore offers a simple, highly attractive alternative to the costly nucleic acid-based HIV infection diagnosis test. In principle, it can be adapted for the detection of any analyte with a suitable antibody, making it a versatile tool for ultrasensitive diagnostics. Despite of great simplicity and ultrasensitivity, the plasmonic ELISA however, has a rather small dynamic range of one order of magnitude, making it difficult to quantify the exact target concentration.

## 2. Conclusion and outlook

Significant advances have been made over the past few years in the development of PCR-free assays suitable for specific and ultrasensitive detection of SNPs. The incorporation of various biofunctionalised nanomaterials coupled with novel amplification strategies have permitted the detection of extremely low concentrations of SNPs, down to the aM–zM range. Such levels of sensitivity have already compared very favorably with many PCR based methods. In general, the amplification strategies may be classified as one of the following categories:

(i) Nanoparticle-target assisted PCR amplification.

(ii) Nanoparticle-target assisted probe amplification.

(iii) Target recycling coupled with probe and/or signal amplification.

(iv) Tandem amplification schemes and signal catalytic cascades.

(v) Nanomaterial enhanced signal amplification.

In general, the sensitivity of a nanomaterial-based SNP assay can be greatly enhanced by combining target, probe and signal amplification schemes. Most of the recent target and probe amplification schemes have exploited the great catalytic power and specificity of enzymes to achieve ultra-sensitivity and specificity. Of particular interest here is the use of DNAzymes that can undergo the so-called enzymatic cascade reactions, where the activation of multiple enzymes by a target DNA can result in ultra-sensitivity, comparable many PCR based assays. Another widely used strategy is the use of restriction enzymes that specifically recognise the restriction sites to degrade the reporter probe, allowing for target recycling. These autocatalytic strategies have resulted in unmatched sensitivities while still maintaining high specificity. However, a limitation here is that restriction enzymes can only recognize a specific sequence and therefore are not suitable for universal SNP detection. Several ingenious ways in the design of nanomaterials for signal amplification strategies have also been developed, including the use of multiple tagging and enrichment of nanoparticle probes with signal moieties to enable ultra-sensitivity. Some of these strategies also hold great promises for multiplexed detection, an important property for high diagnostic accuracy.

Despite significant advances, several limitations still need to be resolved before they can be translated into clinical diagnostic assays. For example, although a number of assays have reported aM, even zM sensitivity, most of which were still at the proof-of-concept stage and were carried out under clean buffers. They have not yet been tested in clinically related media, such as blood, serum, saliva and urine. Furthermore, most assays have reported a rather limited SNP discrimination ratio (<10 fold), making them potentially unsuitable for detection of low abundant disease related SNPs in the background of wild-type gene/genomic DNA because of the strong interference from the background DNAs. Moreover, the lack of the multiplexing capability could pose a significant limiting factor for the real clinical potential. The stability of the biofunctionalised nanomaterials is another important issue for such nano-enabled SNP biosensors. In this regard, the fundamental understanding of the biomolecule-nanomaterial interactions is imperative to alleviate problems of high background signals due to non-specific adsorptions in serum and/or other complex clinical samples.

The use of biofunctionalised nanomaterials together with novel amplification strategies has transcended barriers to the attainment of extremely low detection limits of disease-related SNPs. This ultimately ushers the way for more practical concerns such as the realistic applications of the technology in clinical settings. Even more challenging is the development of a robust, portable, point-of-care diagnostic system that can specifically detect the ultralow level of disease-related SNPs rapidly and conveniently on the site of the patient and in places such as the doctor's office, school clinic and in patients’ residence.^89^

These also call for improving the assays’ amenability towards automation and miniaturization because most current assays still require the use of expensive and complex instrumentation and complex procedures, limiting their potential in rapid diagnosis. Electro-chemical signal transduction can provide an option for the miniaturization and automation but these methods are prone to false positives.^26^ The recent advances in microfluidics^90,91^ may be able to address sample throughput and automation challenges in SNP assays. If all of these challenges were met, the automation of these ultrasensitive assays may lead to the integration of sample processing, quantification and signal measurement in an all-in-one device in real clinical setting. This would greatly facilitate the rapid, accurate disease diagnosis and prognosis. In view of this, these are still crucial challenges that need to be addressed, and more efforts will be needed to improve the analytical sensing performance and portability of SNP assays.
